# Multifocal necrotizing fasciitis: what makes it different? A German trauma center experience

**DOI:** 10.1007/s00068-026-03293-3

**Published:** 2026-08-19

**Authors:** Franziska Keßler, Johannes Frank, Philipp Störmann, Nils Wagner, André El Saman, Ingo Marzi, Maximilian Leiblein

**Affiliations:** Department of Trauma Surgery and Orthopedics, University Hospital, Goethe University, Theodor-Stern-Kai 7, 60590 Frankfurt am Main, Germany

**Keywords:** Necrotizing fasciitis, Multifocal necrotizing fasciitis, Synchronous, Multifocal necrotizing fasciitis, Soft tissue infection, Critical care

## Abstract

**Background:**

Multifocal necrotizing fasciitis (MulNF) is a rare presentation of necrotizing fasciitis (NF) characterized by involvement of more than one non-contiguous anatomical site during the same disease episode. Comparative data between monofocal and multifocal NF remain limited.

**Methods:**

We performed a retrospective single-center cohort study of patients treated for NF between 2015 and 2026. Patients were classified as MNF (single site) or MulNF (multiple non-contiguous sites) based on chart review. Demographics, comorbidities, microbiology, laboratory values, organ failure, ICU course, surgical burden, and outcomes were compared.

**Results:**

Forty-seven patients were included, of whom five (10.6%) had multifocal NF. Four of these showed clearly synchronous non-contiguous involvement, whereas one developed a delayed second non-contiguous focus within the first four days of the same disease episode. Compared with MNF, MulNF was associated with higher ICU burden, more frequent mechanical ventilation, and a greater number of debridements and total operations. Higher rates of septic shock, hemodynamic failure, and renal replacement therapy were observed in MulNF. No deaths occurred in the small multifocal cohort, whereas 11/42 patients with monofocal NF died during hospitalization. Given the small multifocal sample size and multiple comparisons performed, all findings should be interpreted cautiously.

**Conclusion:**

In this single-center cohort, multifocal NF represented a rare but clinically severe disease pattern associated with greater ICU and surgical burden than monofocal NF. These findings support heightened clinical vigilance for additional non-contiguous foci and emphasize the importance of rapid diagnosis and repeated surgical assessment. Larger multicenter studies are needed to validate these exploratory observations and better define the clinical relevance and pathogenesis of multifocal NF.

## Introduction

Necrotizing fasciitis (NF) is an aggressive soft tissue infection with a frequently fulminant course, characterized by necrosis of subcutaneous tissue and fascia [1–4]. NF is life-threatening and associated with high mortality [3–6]. Prognosis depends on early recognition and immediate initiation of appropriate therapy [3, 6].

Diagnosis is primarily clinical and can be challenging because characteristic skin findings are often absent [3, 4, 6, 7]. “Pain out of proportion” is a key warning sign. Imaging and laboratory scores such as LRINEC can support clinical assessment but do not replace it [4, 7, 8]. Management requires urgent surgical debridement combined with broad-spectrum antimicrobial therapy, and aggressive removal of necrotic tissue is critical for survival [3, 6, 7].

From an epidemiological perspective, NF is rare, with incidences typically below two cases per 100,000 inhabitants in industrialized countries [5, 9, 10]. Microbiologically, it can be classified in four types: type I (polymicrobial), type II (usually *Streptococcus pyogenes*), type III (e.g., *Vibrio* spp., *Clostridium* spp., other gram-negative bacteria) and type IV (fungal infection) [3, 4, 11].

NF usually affects a single region, most commonly a limb [11]. However, in rare cases, multiple non-contiguous sites are affected simultaneously.

More recent reports have distinguished synchronous lesions developing within hours from metachronous lesions developing days apart [7, 12, 13]. MulNF appears to be associated with higher mortality than monofocal NF (MNF) [1, 6, 7, 14, 15]. The multifocal pattern has been hypothesized to result from hematogenous dissemination or septic emboli [4], and atypical presentation may further delay diagnosis [4, 15].

Despite the clinical relevance of MulNF, systematic data remain sparse. Most reports are single case studies [7, 13–21], and only a few series exist [1, 4, 22]. Comparative analyses of monofocal and multifocal NF regarding presentation, diagnosis, treatment, and outcomes are therefore limited.

Accordingly, we aimed to analyze MulNF cases treated at our center and to highlight differences compared with monofocal NF.

## Materials and methods

This retrospective single-center study was approved by the institutional ethics committee of the Goethe-Universität Frankfurt (**2026–2944)**.

All patients ≥ 18 years diagnosed and treated for necrotizing fasciitis between January 2015 and January 2026 at our level-one trauma center were screened and retrospectively reviewed. Part of this cohort has been reported previously in a different context [11]. In total, 15 patients treated during the earlier study period overlapped with the previous publication; the present study addresses a distinct research question and includes additional cases and variables not reported previously.

Diagnosis was based primarily on clinical findings (erythema, swelling, edema, and pain out of proportion) and intraoperative findings (fascial swelling and necrosis, loss of fascial integrity, gray discoloration, extensive necrosis, characteristic separation between skin, fascia, and muscle, and “dishwater” pus), with microbiological findings (Gram stain and culture) and, when available, histopathological results used as supportive diagnostic information, in accordance with the diagnostic approach proposed by Zhou et al. [23].

Patients were stratified into multifocal necrotizing fasciitis (MulNF) and monofocal necrotizing fasciitis (MNF). MulNF was defined as involvement of more than one non-contiguous anatomical site without spread per continuitatem during the same disease episode. Within the MulNF group, cases were classified descriptively as synchronous when additional foci were present at admission or became apparent within hours, and as metachronous when a second non-contiguous focus developed after a delay of days during the same disease episode.

Demographics (age, sex), comorbidities (diabetes, chronic kidney disease, liver cirrhosis, malignancy, immunosuppression, intravenous drug abuse, peripheral arterial disease), and ASA class were recorded.

Disease course and management were assessed by anatomical site, extent of spread and etiology. The presumed portal of entry was determined retrospectively from the medical record based on the clinically most likely local source of infection. Documented skin lesions, wounds, ulcers, recent surgical sites, bites, or injection/IV access at the affected anatomical region were classified accordingly; if no plausible source was identifiable, the portal of entry was coded as unknown.

Isolated pathogens of the first samples taken and antibiotic regimens were reviewed. The first available laboratory values were used to calculate the LRINEC score.

Timing to first surgical debridement, length of hospital and ICU stay and presence of septic shock were documented. Organ dysfunction variables were assigned retrospectively from chart review using predefined clinical criteria. Respiratory failure was assessed by impaired oxygenation (Horovitz ratio, where available) and/or the need for mechanical ventilation, hemodynamic failure by norepinephrine requirement, hepatic failure by bilirubin elevation and impaired synthetic liver function, and renal failure by creatinine, need for renal replacement therapy, and KDIGO-based acute kidney injury staging. Multiorgan failure was recorded when more than one organ system was affected during the same disease episode.

Operative burden (number of debridements and total operations) and outcomes (limb salvage, reconstructive procedures and mortality) were analyzed. In-hospital mortality was the primary endpoint.

All statistical analyses were exploratory and descriptive rather than confirmatory. No multivariable modeling was performed, as such approaches would not have been robust in this sample size.

Continuous variables are reported as median and interquartile range (IQR), and categorical variables as counts and percentages. Between-group comparisons between monofocal necrotizing fasciitis (MNF) and multifocal necrotizing fasciitis (MulNF) were performed using the Mann-Whitney U test for continuous variables and Fisher’s exact test for categorical variables. Effect estimates were calculated to describe the magnitude and direction of observed differences, but all results were interpreted as hypothesis-generating given the very small MulNF sample size and the large number of comparisons. No formal correction for multiple testing was applied. Figures were created in R (base graphics).

## Results

Given the exploratory design and the very small number of MulNF cases, all between-group comparisons should be interpreted descriptively and with caution. The analyses were intended to identify clinically relevant patterns rather than to provide confirmatory evidence of group differences.

### Demographic data

Between January 2015 and January 2026, a total of 47 patients were treated for necrotizing fasciitis in our clinic. Of those, 42 were diagnosed with monofocal (MNF) and five with multifocal necrotizing fasciitis (MulNF). Among the five patients classified as having multifocal disease, four showed clearly synchronous non-contiguous involvement with multiple foci already present at admission or becoming apparent within a few hours, whereas one patient with lower-leg and scrotal involvement developed a second non-contiguous focus after a delay of four days and was therefore classified as metachronous within the same disease episode.

Of the patients with MNF 36 were male and six female (85.7% vs. 14.3%), in the group with MulNF three were male and two female (60% vs. 40%).

**Fig. 1:**
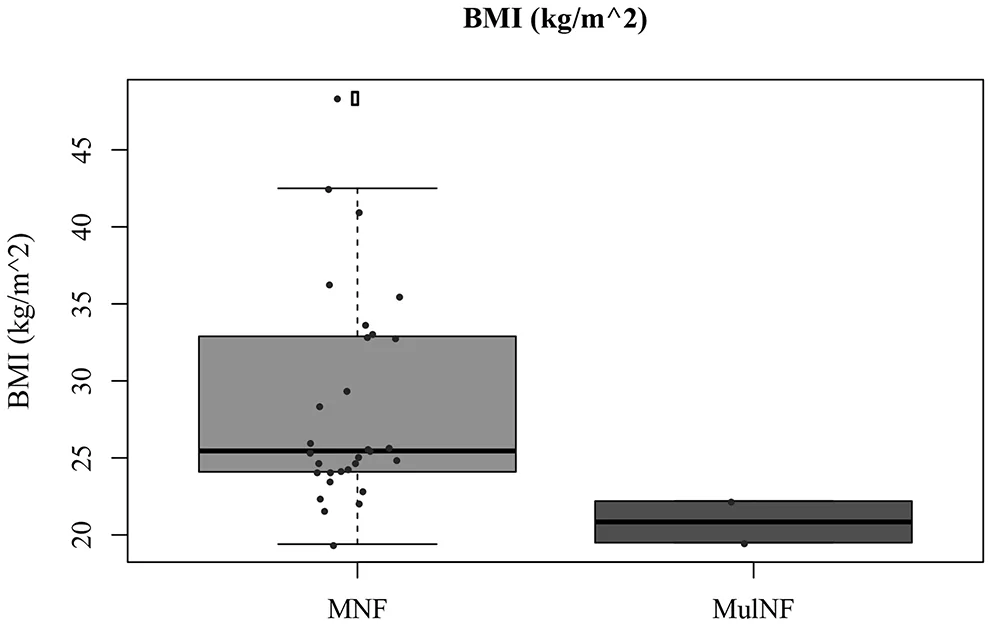
Distribution of body mass index (BMI, kg/m²) in patients with monofocal (MNF) versus multifocal necrotizing fasciitis (MulNF). Boxplots display median and interquartile range; points represent individual patients. BMI was lower in the MulNF group

Patients with MNF were older than patients with MulNF (MNF: 49.5 [41.0–65.25] years, MulNF: 41.0 [41.0–44.0] years). Body mass index appeared higher in MNF compared to MulNF (MNF: 25.45 [24.13–32.88] kg/m^2^, MulNF 20.85 [20.18–21.52]) (Fig. 1).

In terms of presentation type, MNF cases were more often primary presentations (29/42, 69%) than secondary transfers (13/42, 31%), while MulNF cases showed a similar pattern with three (60%) primary presentations and two (40%) secondary transfers.

### Comorbidities

The following comorbidities were evaluated and compared between patients with MulNF and MNF (Table 1).

In our cohort, diabetes was present in 25.5%, peripheral arterial disease in 4.3% and chronic kidney disease in 2.1%. In addition, intravenous drug abuse was present in 17.0% (MNF 6/42, 14.3%, MulNF 2/5, 40%) of patients, malignancy in 8.5% and immunosuppression in 4.3%. Comorbidities were broadly similar between patients with MNF and MulNF.

**Table 1:** Comorbidities in patients with monofocal (MNF) and multifocal necrotizing fasciitis (MulNF), presented as absolute numbers and percentages

Variable	MNF *n*/*N* (%)	MulNF *n*/*N* (%)
diabetes	11/42 (26.2%)	1/5 (20.0%)
ckd	0/42 (0.0%)	1/5 (20.0%)
cirrhosis	2/42 (4.8%)	0/5 (0.0%)
malignancy	4/42 (9.5%)	0/5 (0.0%)
immunosuppression	2/42 (4.8%)	0/5 (0.0%)
IVDA	6/42 (14.3%)	2/5 (40.0%)
Peripheral arterial disease	2/42 (4.8%)	0/5 (0.0%)

Of the patients with MulNF two (40%) had an ASA-score of III, two (40%) of IV and one (20%) of V. Of the patients with MNF ASA score I was documented in one patient (2.6%), II in 10 patients (25.6%), III in 13 patients (33.3%), IV in eleven patients (28.2%) and V in four patients (10.3%). In three patients, no ASA-score was documented.

### Locations and etiology

Affected site in patients with MNF was arm in six patients, leg in 33 patients and trunk in three patients.

Among the MulNF cases, the involved sites were as follows: one patient had left foot/lower-upper leg plus right hand/forearm; one patient had lower leg plus scrotum; one patient had bilateral lower- and upper-leg involvement plus right forearm/upper arm; one patient had bilateral upper extremities plus the left leg; and one patient had left gluteal/upper-leg involvement plus the right forearm/upper arm.

Across the entire cohort, the presumed portal of entry was most often unknown (15/47, 31.9%), followed by skin lesion (9/47, 19.1%), injection/IV access (8/47, 17%), trauma (7/47, 14.9%), ulcer (5/47, 10.6%), surgical wound (2/47, 4.3%), and insect bite (1/47, 2.1%); descriptively, MulNF cases clustered around injection/IV access (3/5, 60%) and unknown (2/5, 40%), whereas MNF showed a broader distribution across all categories, IV-access and injection were documented in 5/42 patients (11.9%).

### Laboratory results

**Figs. 2–3:**
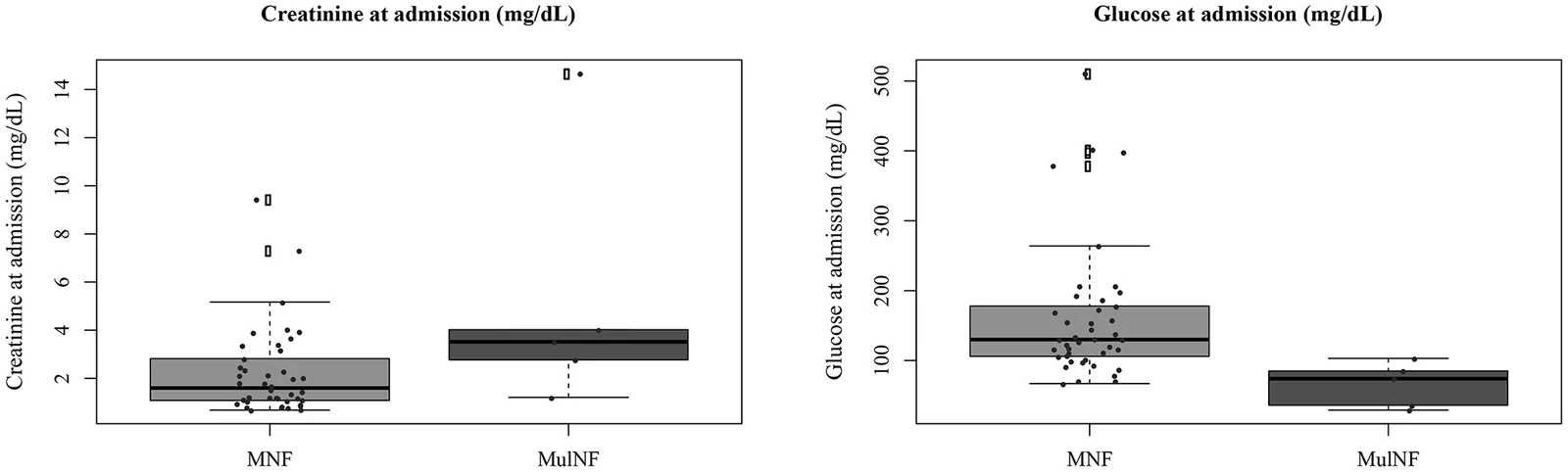
Distribution of creatinine at admission (Fig. 2, mg/dl) and glucose at admission (Fig. 3, mg/dl) in patients with monofocal (MNF) versus multifocal necrotizing fasciitis (MulNF). Boxplots display median and interquartile range; points represent individual patients. Admission creatinine was higher in MulNF, whereas admission glucose was lower in the MulNF group

Laboratory values at admission were largely comparable between groups. The LRINEC score did not differ between MulNF and MNF (median 8.00 [IQR 7.00–9.00] vs. 8.00 [6.00-9.75]). Creatinine levels were higher and Glucose levels lower in the MulNF-group than in MNF group (Figs. 2 and 3).

Table 2 shows key exploratory laboratory findings at admission in patients with monofocal (MNF) and multifocal necrotizing fasciitis (MulNF).

**Table 2:** Key exploratory laboratory findings at admission in patients with monofocal (MNF) and multifocal necrotizing fasciitis (MulNF). Values are presented as median [IQR]

Variable	MNF median [IQR]	MulNF median [IQR]
LRINEC score	8.00 [6.00-9.75]	8.00 [7.00–9.00]
Creatinine at admission (mg/dL)	1.60 [1.08–2.73]	3.52 [2.77–4.02]
Glucose at admission (mg/dL)	130.00 [106.25-176.75]	74.00 [36.00–85.00]
Lactate at admission (mg/dL)	28.50 [19.50-55.25]	23.00 [13.00–41.00]

### Organ failure and Intensive care therapy

All MulNF patients required mechanical ventilation, compared with 45% of MNF patients. MulNF patients showed higher rates of septic shock (100% vs. 47.6%), hemodynamic failure (100% vs. 54.8%), and need for renal replacement therapy (80.0% vs. 33.3%). Ventilation duration tended to be longer in patients with MulNF (median 9.5 [5.25–20.25] days) than in patients with MNF (3.0 [1.0–7.0] days). Rates of renal failure, hepatic failure, respiratory failure, and multi-organ failure were broadly similar between groups.

LRINEC score showed no clear relationship with the development of multi-organ failure or failure of one single organ system.

**Figs. 4–5:**
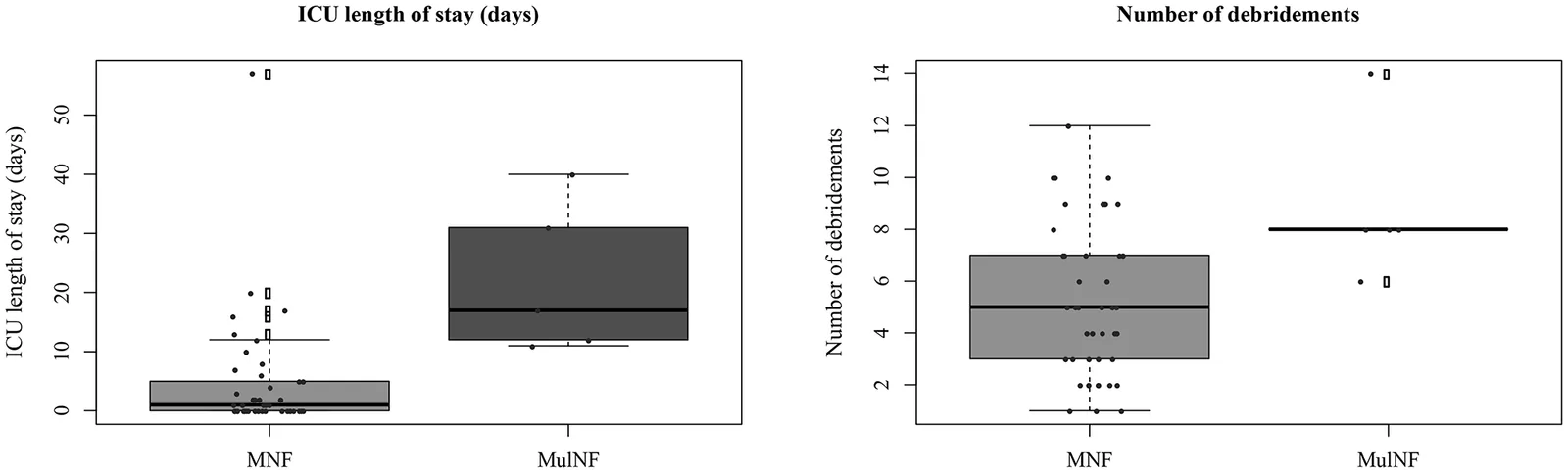
ICU length of stay (Fig. 4, days) and number of debridements (Fig. 5) in patients with monofocal (MNF) versus multifocal necrotizing fasciitis (MulNF). Boxplots display median and interquartile range; points represent individual patients. ICU stay and the number of debridements were higher in the MulNF group

ICU length of stay was longer in MulNF patients than in MNF patients (17.0 [12.0–31.0] vs. 1.0 [0.0–5.0] days) (Fig. 4), whereas overall hospital length of stay was longer in MulNF (56.0 [37.0–93.0] vs. 26.5 [13.5–47.0] days).

ICU admission was required in 24/42 patients with MNF (57.1%) and in all patients with MulNF (5/5, 100%). Accordingly, the shorter ICU length of stay in the MNF group partly reflects that 42.9% of MNF patients did not require intensive care treatment at all.

MulNF patients underwent more debridements (median 8.0 [8.0–8.0] vs. 5.0 [3.0–7.0]) (Fig. 5) and total operations (8.0 [8.0–9.0] vs. 6.0 [3.25–8.0]), whereas rates of amputation, VAC therapy, skin grafting, and flap surgery were broadly similar between groups.

### Microbiology and antibiotics

Blood cultures were positive in 40.7% of MNF patients and in 60% of MulNF patients.

Microbiologically, type I (polymicrobial) and type II (monomicrobial with A-streptococcus) NF predominated in both groups, but type II disease was more frequent in MulNF (60% vs. 43.6%). In total, S. pyogenes (Lancefield group A) was identified in 28 patients and was the most frequent pathogen overall, followed by S. aureus in 12 patients. Presumed injection- or IV access-related portals of entry were more frequent in MulNF (60%) than in MNF (11.9%), and intravenous drug abuse was also more common in MulNF (2/5, 40% vs. 6/42, 14.3%).

### Outcome and mortality

No deaths occurred in the small MulNF cohort, whereas 11/42 patients with MNF died during hospitalization (26.2%). However, given the very small MulNF sample size, no reliable between-group inference regarding mortality can be made. Limb loss was slightly more frequent in MulNF than in MNF (20.0% vs. 17.5%).

Time from admission to first operation was shorter in MulNF than in MNF (355 [140–367] vs. 569 [257–976] minutes).

Key descriptive differences between patients with MNF and MulNF are summarized in Table 3.

**Table 3:** Key exploratory differences between monofocal (MNF) and multifocal necrotizing fasciitis (MulNF). Continuous variables are presented as median [IQR] and categorical variables as n/N (%)

Variable	MNF	MulNF
BMI (kg/m²)	25.45 [24.13–32.88]	20.85 [20.18–21.52]
Mechanical ventilation, n/N (%)	18/40 (45.0%)	5/5 (100.0%)
Septic shock, n/N (%)	20/42 (47.6%)	5/5 (100.0%)
Renal replacement therapy, n/N (%)	13/39 (33.3%)	4/5 (80.0%)
ICU length of stay (days)	1.0 [0.0–5.0]	17.0 [12.0–31.0]
Number of debridements	5.0 [3.0–7.0]	8.0 [8.0–8.0]
Total number of operations	6.0 [3.25-8.0]	8.0 [8.0–9.0]
Hospital length of stay (days)	26.5 [13.5–47.0]	56.0 [37.0–93.0]
Total antibiotic duration (days)	21.5 [12.5-33.75]	48.0 [37.0–49.0]

## Discussion

Necrotizing fasciitis is a rapidly progressive, life-threatening infection of the fascia associated with substantial morbidity and mortality [24, 25].

The most commonly affected sites are the lower and upper extremities, followed by the anogenital region and trunk [3, 25, 26]. Typically, the disease presents monofocal and spreads contiguously [7, 16]. In contrast, synchronous / metachronous multifocal necrotizing fasciitis (MulNF) involves multiple non-contiguous sites and has been reported with higher mortality than monofocal disease (MNF) [22]. The objective of this study was to compare MulNF and MNF with respect to pathogenesis, clinical presentation, treatment, and outcomes.

Although necrotizing fasciitis is often considered within the broader spectrum of necrotizing soft tissue infection (NSTI), our analysis focused on cases with clinical and intraoperative findings consistent with necrotizing fasciitis.

Our findings suggest that multifocal necrotizing fasciitis may represent a distinct clinical phenotype characterized by either hematogenous dissemination or repeated local inoculation, leading to a more severe systemic course.

MulNF accounts for approximately 5 − 13% of NF cases in the literature [1, 16, 22]. In line with this, 10.6% of patients in our cohort exhibited multifocal non-contiguous disease.

The most frequent comorbidities were diabetes mellitus and intravenous drug abuse (IVDA). IVDA was more common in MulNF (40%) than MNF (14.3%). Diabetes is the most common comorbidity in NF [27]. Nasu et al. suggested diabetes as a risk factor for MulNF in addition to liver cirrhosis and end-stage renal disease [21]; in our cohort, cirrhosis and ESRD were not more prevalent. According to the Robert-Koch-Institute, in the German adult population, the prevalence of diagnosed diabetes is about 7.2% (ages 18–79), which is substantially lower than in our NF cohort, confirming diabetes as a risk factor for NF in general rather than specifically for MulNF.

Among MulNF patients, four had simultaneous involvement of upper and lower extremities and one had lower-leg plus scrotal involvement. Blood cultures were positive in 40.7% of MNF patients and in 60% of MulNF patients. While the literature often hypothesizes hematogenous dissemination by highly virulent pathogens as a cause of multifocality [16, 22], our data suggest that no single mechanism explains all multifocal cases. Even in large NF series, blood cultures are positive in only about 20% of cases [28], so the absence of an even higher bacteremia rate in our small MulNF sample may simply reflect the generally low yield of blood cultures. One multifocal case in our cohort was more appropriately classified as metachronous than strictly synchronous, with lower-leg and scrotal involvement becoming clinically apparent four days apart. In analogy to Guzmán et al. [12], who used the term metachronous for a comparable delay, this classification appears appropriate. In that patient, positive blood cultures, the absence of intravenous drug abuse, and diabetes mellitus as the major underlying comorbidity make hematogenous dissemination more plausible than a second independent inoculation event, although this remains speculative. At the same time, MulNF cases in our cohort clustered around injection-/IV-access-related portals of entry, and IVDA was more common than in MNF. These findings support repeated local inoculation as an alternative mechanism in at least part of the cohort.

Microbiologically, type II disease was more frequent in MulNF, and S. pyogenes was common, whereas marine Gram-negative pathogens reported in some coastal Asian cohorts were absent [22]. Type-II NF is considered more aggressive and is associated with bacteremia and toxic shock, which may increase the likelihood of multifocal disease through septic embolic or toxin-mediated mechanisms [4, 29]. Superantigen-driven cytokine release and endothelial injury provide a biologically plausible substrate for rapid or multifocal progression [23, 29]. Microvascular injury and thrombosis described in NF could facilitate multifocal progression and may also limit detectable bacteremia, but this remains speculative and should be tested prospectively [16, 23].

Taken together, these observations support the concept that multifocal NF is etiologically heterogeneous and may arise through different pathogenetic pathways depending on host background, portal of entry, and pathogen spectrum.

Admission laboratory values were analyzed for all patients. The LRINEC score [8] did not differ between groups but was positive (≥ 6 points) in both (MulNF 8 [7–9] vs. MNF 8 [6-9.75]). Admission creatinine showed an increase in MulNF compared with MNF, consistent with prior NF literature identifying elevated creatinine as a marker of disease severity and adverse outcomes [10, 30]. Thus, higher creatinine in MulNF may reflect more severe systemic illness rather than a pathogen-specific effect. Admission glucose was lower in MulNF than in MNF. In contrast to NF literature emphasizing stress hyperglycemia or the glycemic gap as severity markers in diabetic NF [27], our MulNF patients showed lower admission glucose values. Given the very small MulNF sample size, no firm explanation can be derived; possible mechanisms include greater metabolic consumption or sepsis-related dysregulation.

In our cohort, MulNF patients required more intensive care resources than MNF patients. Mechanical ventilation was more frequent and ICU length of stay was markedly longer. These findings align with published cohorts, that consistently report greater severity, including higher rates of shock, fulminant sepsis, organ dysfunction and a more rapid onset of symptoms [22].

We also observed higher rates of septic shock, hemodynamic failure and renal replacement therapy in MulNF, and a greater operative burden with more debridements and total operations. These observations support the clinical impression that multifocal disease represents a more severe systemic phenotype.

Unexpectedly, no deaths occurred in the small MulNF cohort. This contrasts with the much higher mortality reported in other MulNF cohorts [16, 22] and must be interpreted in the context of the small MulNF sample size.

Several factors could have been contributing to this discrepancy: MulNF patients were slightly younger than MNF patients, which may have conferred greater physiologic reserve. Admission-to-debridement times were shorter in MulNF than in MNF patients (355 vs. 569 min), which is associated with improved outcomes [5].

The pathogen spectrum differed from high-mortality series. Other MulNF cohorts report a high proportion of marine Gram-negative pathogens, particularly *Vibrio* spp., coming along with characteristic comorbidities and being associated with fulminant disease and high mortality [1, 31, 32, 33]. In contrast, *S. pyogenes* was common in our cohort (in all MulNF patients) and marine Gram-negative pathogens were not observed, likely reflecting geographic differences. However, the small MulNF sample size limits the ability to detect mortality differences and may mask true outcome disparities.

Taken together, our findings support a more aggressive diagnostic and surgical approach in suspected multifocal necrotizing fasciitis, including early comprehensive whole-body assessment, a low threshold for repeated surgical exploration, and heightened clinical vigilance, particularly in patients with intravenous drug use or multiple potential portals of entry.

### Limitation

This study has several limitations. Its retrospective, single-center design and the very small MulNF sample size limit generalizability and statistical inference. Some variables could not be consistently or completely extracted from retrospective records, resulting in missing data for several analyses. In addition, a relatively large number of comparisons was performed without formal correction for multiple testing, increasing the risk of type I error. The reported between-group analyses should therefore be interpreted as exploratory and descriptive, particularly for findings close to the conventional significance threshold. Finally, no multivariable modeling or matching approach was performed, so observed associations may be confounded.

Part of the cohort overlapped with a prior publication from our center [11], but the current analysis focuses on multifocal NF and provides new comparisons and outcomes.

## Conclusion

In this 11-year single-center cohort, MulNF accounted for 10.6% of NF cases and was associated with greater disease severity, reflected by higher ICU burden, more surgical interventions, and higher rates of mechanical ventilation.

No deaths occurred in the small MulNF cohort. This finding should be interpreted cautiously and may reflect early surgical management, younger age, and a pathogen spectrum differing from high-mortality cohorts dominated by marine Gram-negative pathogens. Clinically, these findings highlight the need for heightened vigilance for multifocal disease, especially in patients after injection, IV access or IVDA, and reinforce the importance of rapid diagnosis, repeated clinical assessment for additional foci, and aggressive, repeated debridement. Given the exploratory design, small MulNF sample size, and multiple comparisons performed, these findings should be interpreted cautiously and require validation in larger multicenter cohorts to better define MulNF pathogenesis and clinical relevance.

## Data Availability

Data available on request.

## Declarations

### Declarations of generative AI use

During the preparation of this manuscript, the authors used AI-assisted language tools for editing and language refinement. The authors reviewed and edited all AI-generated suggestions and take full responsibility for the content of the published article.
